# Supplementary material for: OVOL2 induces mesenchymal-to-epithelial transition in fibroblasts and enhances cell-state reprogramming towards epithelial lineages
Source: Sci Rep. 2019 Apr 24;9:6490. doi: 10.1038/s41598-019-43021-z (PMC6482152; doi:10.1038/s41598-019-43021-z)
Supplement: Supplementary file 1 — Supplementary information [file 41598_2019_43021_MOESM1_ESM.pdf]

Manuscript title

OVOL2 induces mesenchymal-to-epithelial transition in fibroblasts and enhances cell-state reprogramming towards epithelial lineages

Kazuhide Watanabe[1,*], Ye Liu[1], Shuhei Noguchi[1], Madeleine Murray[1], Jen-Chien Chang[1], Mami Kishima[1], Hajime Nishimura[1], Kosuke Hashimoto[1], Aki Minoda[1], and Harukazu Suzuki[1,*]

[1] RIKEN Center for Integrative Medical Sciences, 1-7-22 Suehiro-cho, Tsurumi-ku, Yokohama, Kanagawa 230-0045, Japan

* To whom correspondence should be addressed. Tel: +81-45-503-9222; Fax: +81-45-503-9216; Email: harukazu.suzuki@riken.jp Correspondence may also be addressed to kazuhide.watanabe@riken.jp

SUPPLEMENTAL TABLES

Table S1. List of E-, K-, M-, and F-specific marker genes

Provided as an Excel file

Table S2. Accessibility score of TF motifs

Provided as an Excel file

Table S3. Primers used in this study

**Target gene**	Forward primer	Reverse primer
CDH1	AAAGGCCCATTTCCTAAAAACCT	TGCGTTCTCTATCCAGAGGCT
VIM	GACGCCATCAACACCGAGTT	CTTTGTCGTTGGTTAGCTGGT
KRT14	GCGGCCTGTCTGTCTCAT	TGAGCCGCATTCTGAACGAG
TP63(endogenous)	GGTCCCCACAGAGCAAGA	TGCAATGACAGCCCTTGA

SUPPLEMENTAL FIGURE LEGENDS

Figure S1. Correlation between the expression of representative E and M markers.

(**A**) Analysis with CCLE datasets. Pearson correlation coefficient r was calculated between each transcript and representative E genes (CDH1, EPCAM, KRT19, and TJP1) and M genes (VIM, MMP3, CDH2, and FN1) across 1038 microarrays for cancer cell lines. The correlation coefficient r for each transcript between the indicated combinations of E and M markers is shown in the scatter plots. (**B**) Analysis with FANTOM5 datasets. Expression correlation was analyzed across 1829 CAGE data for normal cells, as described in (**A**).

Figure S2. Expression correlation analysis of FANTOM5-defined TFs with FANTOM5 CAGE datasets.

Among 1995 FANTOM5-defined TFs, 1675 TF transcripts were identified in the FANTOM5 datasets. The Pearson correlation coefficient *r* was calculated between each transcript and *CDH1* or *VIM* across 1829 FANTOM5 CAGE data for normal cell types. Representative EMT-TFs (blue) and 16 candidate MET-TFs (red) were labeled.

Figure S3. Heatmap and unsupervised clustering by CAGE and ATAC-seq.

Heatmap with unsupervised clustering of the indicated samples and annotated marker genes for E, M states and keratinocytes by CAGE (**A**) and ATAC-seq (**B**). The top differentially expressed genes and differentially regulated ATAC-seq peaks between control fibroblasts (Control) and primary keratinocytes were used for the analysis. Note that all replicate pairs are co-aggregated at the first level of the dendrogram and the TK + OVOL2 samples changed their expression pattern toward keratinocytes.

Figure S4. Changes in motif accessibility and expression of TFs during reprogramming (ZEB1 and ID3).

Motif accessibility and TF genes expression. Motif enrichment score (*Z* score) and CAGE expression (logTPM) are shown in the top and bottom panels, respectively, for the indicated TFs.

Figure S1

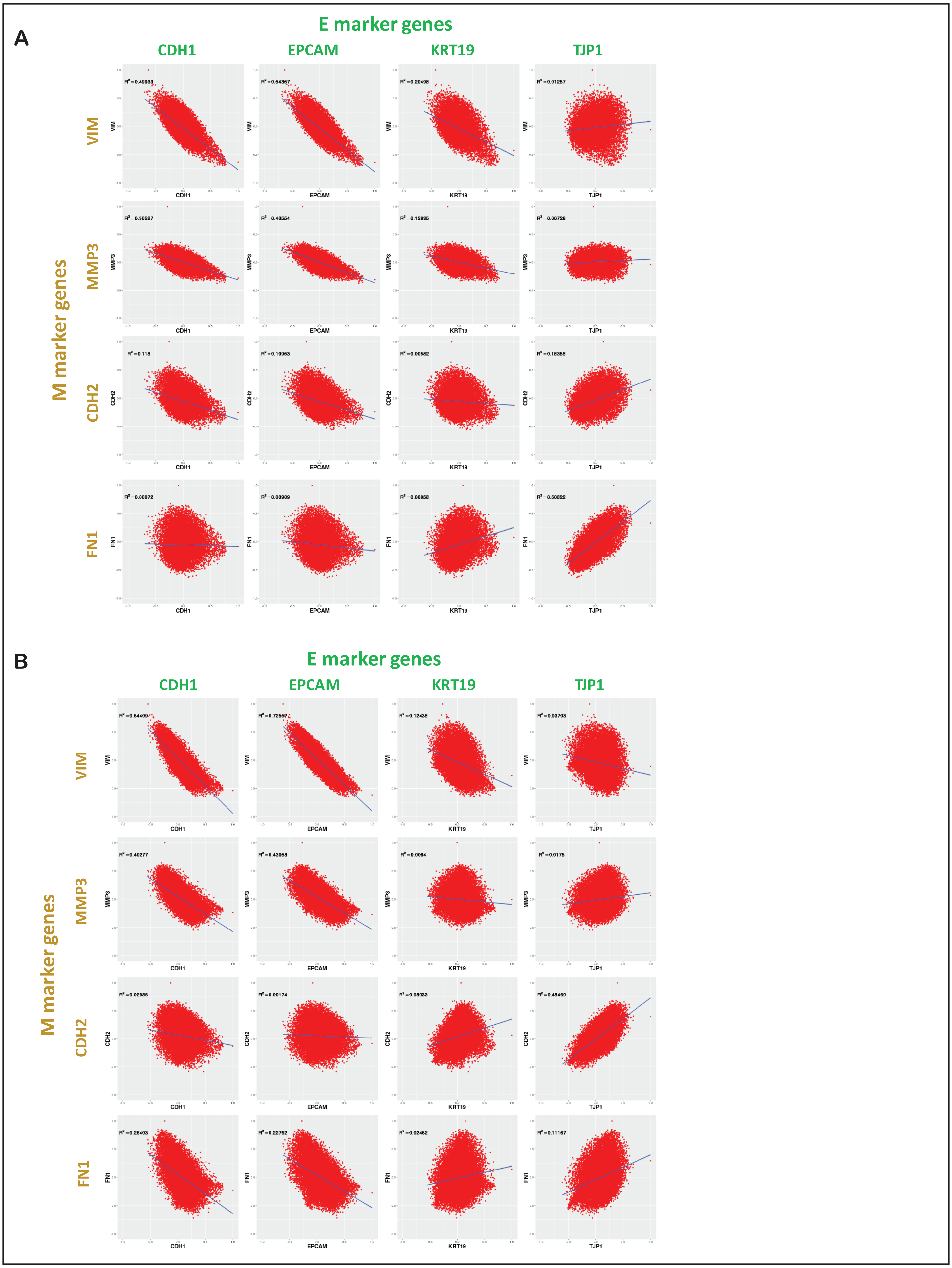
A
E marker genes
CDH1
EPCAM
KRT19
TJP1
M marker genes
VIM
MMP3
CDH2
FN1
B
E marker genes
CDH1
EPCAM
KRT19
TJP1
M marker genes
VIM
MMP3
CDH2
FN1

Figure S2

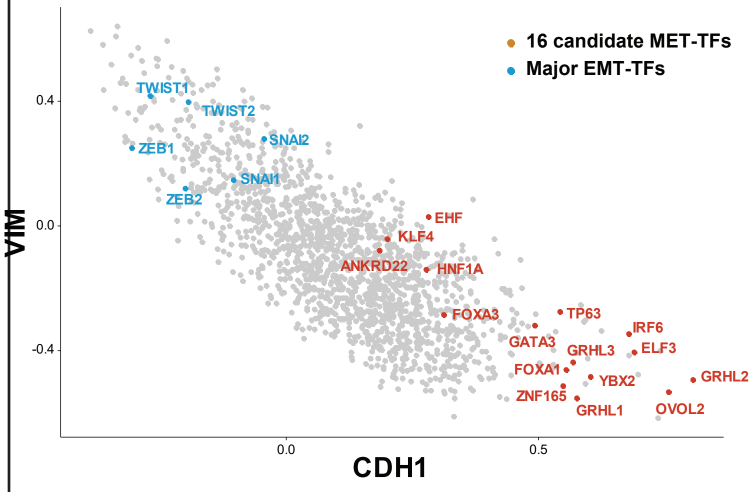

16 candidate MET-TFs
Major EMT-TFs
VIM
CDH1
0.4
0.0
-0.4
0.0
0.5
TWIST1
TWIST2
SNAI2
ZEB1
SNAI1
ZEB2
EHF
KLF4
ANKRD22
HNF1A
FOXA3
TP63
IRF6
GATA3
GRHL3
ELF3
GRHL2
FOXA1
YBX2
ZNF165
GRHL1
OVOL2

Figure S3

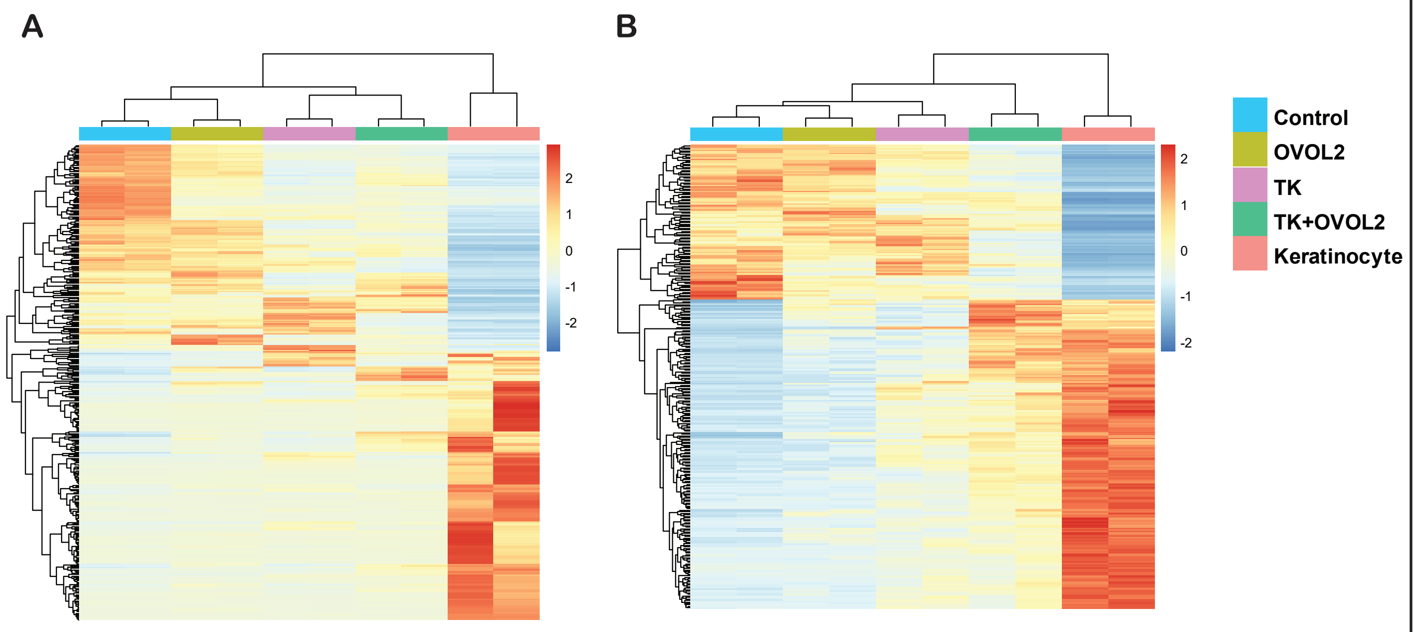

Figure S4

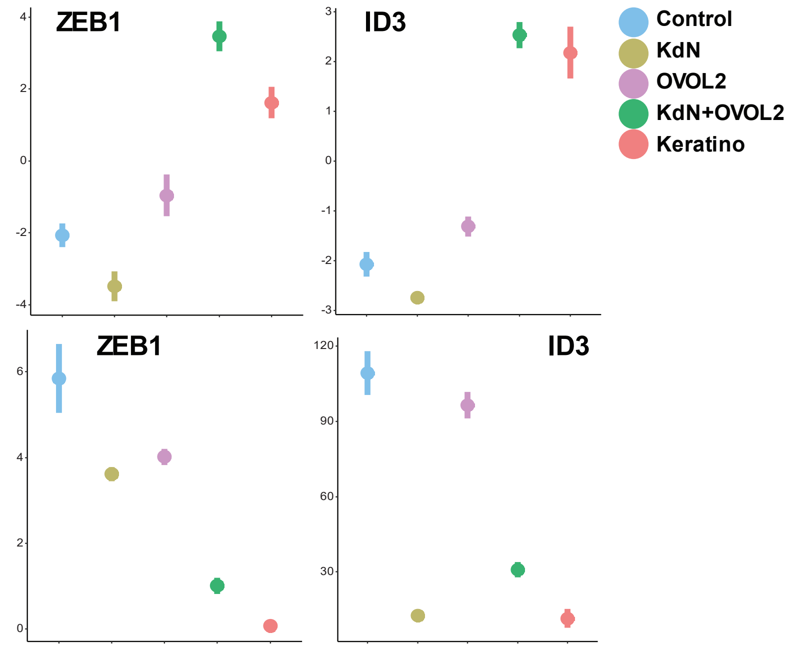
ZEB1
ID3
Control
KdN
OVOL2
KdN+OVOL2
Keratino
4
2
0
-2
-4
3
2
1
0
-1
-2
-3
ZEB1
ID3
6
4
2
0
120
90
60
30
